# Learning and Crises in Human Development. A Sociocultural and Dialogical Approach

**DOI:** 10.1007/s12124-025-09940-5

**Published:** 2025-12-10

**Authors:** Nathalie Muller Mirza

**Affiliations:** Faculty of Psychology & Education, University of Geneva, Geneva, Switzerland

**Keywords:** Adult learning, Sociocultural psychology, Crisis, Human development, Dialogical analysis

## Abstract

This paper examines the relationship between human development, crisis, and learning from a sociocultural and dialogical approach in psychology. In this perspective, we conceptualize crises as ruptures that may arise from external events, interpersonal dynamics, or internal tensions, and are understood not solely as obstacles but as potential conditions for development. Within this framework, learning is approached as a situated, semiotically mediated process of meaning-making, embedded in relational, cultural, and sociohistorical contexts. By integrating biographical and narrative approaches with sociocultural and dialogical theories, this study highlights crisis as a key analytical entry point for investigating learning across the adult life course. The paper presents the results of a dialogical analysis that draws on a longitudinal diary of a woman spanning two decades, offering unique access to the evolving processes of adult learning across life transitions. By examining what we call the “dialogical tensions”, we show how they are related to various spheres of experience, including professional life, family life, health, and spirituality, and articulate personal aspirations and social expectations, as well as the relationships between self and others, past and future selves. These tensions function as critical junctures where the self is reconfigured, fostering development.

## Introduction: Understanding Learning as a Lifelong, Situated, and Dialogical Process

Sociocultural research in developmental psychology has identified three main ways in which “crises” may affect individual thinking and learning. First, crises can manifest as external events – for example, a global pandemic that shuts down educational institutions, or a war that forces someone to abandon their profession and retrain in a new country. Such events interrupt and reshape the environment in which individuals construct their knowledge and lives. Second, crises may be interpersonal: through interaction and argumentation with others, individuals are led to revisit their perspectives, transform their understanding of a topic, or adopt new positions. Third, crises may stem from internal emotional or motivational conflictual dynamics, which play a role in learning processes.

This body of work has produced important insights into how ruptures, transitions, and transformations impact learning across macro-, meso-, and micro-levels of analysis. In this framework, the concept of *crisis* is understood as a lived experience, situated within a relational and sociohistorical context, and potentially generative of change and development (Bernal Marcos et al., 2024; Gillespie et al., 2023). A crisis – a highly polysemous concept in the social sciences (Fassin, 2023; Morin, 1976) - may be experienced as a sudden and unexpected event, or as a longer-term process with cumulative and resonant effects. Learning is here conceived as a situated process, co-constructed in relational and cultural contexts, and fundamentally shaped by meaning-making activities.

Inspired by the work of scholars such as Lev Vygotsky, Jerome Bruner or Michael Cole, sociocultural research has traditionally focused on learning among children and young adults in formal educational contexts, often over relatively short time frames. In contrast, learning in adulthood, particularly over the life course, has received comparatively little attention.

Yet this domain raises critical theoretical and methodological challenges, such as how to: (1) Conceptualize learning as both an intrapsychic *and* situated process. On one hand, it involves personal meaning-making that transforms one’s way of being in the world; on the other, it is shaped and constrained by external events and broader sociopolitical dynamics; (2) Account for the *dynamic*, interpersonal, and processual nature of learning, which unfolds over time since knowledge is constructed, shared, validated or at times disqualified through interaction with others, and (3) Recognize the interconnection between *multiple spheres of experience*, such as family, work, education, and civic engagement, which serve as contexts where knowledge is produced, exchanged, and reconfigured (Zittoun & Gillespie, 2016).

In this chapter, we aim to explore theoretical and methodological directions that extend the sociocultural approach by incorporating insights from *dialogical perspectives*. By conceptualizing the social and the other as internal to the self, dialogical approaches offer a distinct lens through which to analyze crisis phenomena - not as external triggers of internal reaction, but as *tensions manifesting in discourse*, reflecting dialogues that may drive learning and transformation.

This theoretical orientation builds upon critiques of so-called monological theories of mind (Linell, 2009; Marková, 2006), which explain thought primarily as an intrapsychic activity. Dialogical theories, in contrast, view psychological and learning processes as inherently *relational*, constituted through interactions with others and with the social world (Muller Mirza & dos Santos Mamed, 2019, 2021). In this paper, we argue that a sociocultural and dialogical approach in psychology provides valuable conceptual and analytical tools for understanding adult learning throughout the lifecourse.

Our starting point is a unique empirical corpus: a personal diary written over more than twenty years. This longitudinal, self-authored material written by a woman offers a rare opportunity to trace the evolution of meaning-making and learning as expressed in a personal narrative, continuing the lines of inquiry developed by Zittoun, Gillespie, and others.

To this end, we proceed in three stages. We begin by revisiting key contributions from research on adult learning trajectories, particularly from biographical and narrative perspectives. While these works do not always explicitly adopt a sociocultural stance, we argue that they provide useful insights into the relationship between crisis and thinking. We then introduce central tenets of a sociocultural and dialogical approach to learning, which complement life-course studies by focusing more explicitly on the psychological processes involved in meaning-making and transformation. Finally, we present an analysis of selected diary excerpts authored by a woman, whom we will refer to as Applegirl, the pseudo she uses in her diary, focusing on moments in which her writing reflects dialogical tensions. These tensions, we suggest, function at times as *crises* or turning points, emerging at the intersection of different spheres of experience, family, work, and health, and giving rise to new understandings of self, others, and the world.

In addition to contributing to our theoretical discussion, these data allow for reflection on the trajectory of a woman in the contemporary US society, and of the methodological challenges of studying learning and development over time.

## Lifelong Learning Trajectories: Contributions from Biographical and Narrative Approaches

In many theories of learning and cognition, knowledge and learning are often conceptualized as phenomena located within the individual, with the site of learning understood to be behavior or the brain (Resnick, Säljö, et al. 1997). Much research on learning has also focused on formal institutional contexts, neglecting the rich and diverse learning opportunities that take place beyond the boundaries of education systems and in the everyday lives of adults. Additionally, the dynamic and longitudinal dimensions of learning have rarely been examined. In contrast, biographical and narrative approaches have offered important insights into how life trajectories can be understood not merely as the outcomes of external social forces, but as the result of ongoing interaction and sense-making by individuals as actors and authors of their lives.

The 1970 s marked a significant turning point in contemporary societies. Whereas adult life had previously been conceived as relatively linear and predictable, concepts such as rupture, transition, bifurcation, and discontinuity began to characterize life paths in new and profound ways (Spini et al., 2017). The once-synchronized milestones of adulthood became increasingly desynchronized, fragmented, and uncertain, reflecting broader temporal shifts and the growing instability of social frameworks.

In this context, the “adult” emerged not simply as the endpoint of development or the normative standard by which children’s development was measured, but as a dynamic subject of inquiry. Adult life came to be seen as plural and complex, shaped by uncertainty, transformation, and crisis (Baltes, et al. 2006; Bidart, 2006). The traditional life-course model that previously structured adult pathways no longer applied in a universal or prescriptive manner. Increasingly, adults were expected to define their own models of development, navigating uncertain paths with limited institutional guidance or guarantees.

The idea of a “learning society” gained traction during this period, both as an emancipatory vision and as a political and economic imperative. Lifelong learning became a key term in policy discourse, especially in contexts marked by globalization, economic restructuring, and social inequality.

In both French- and English-speaking academic traditions, researchers working with adults and youth affected by unemployment, precarious work, or lack of formal qualifications developed a dual focus: a *critical* analysis of the school system – often seen as exclusionary – and a *theoretical* inquiry into the nature of adult learning (Dominicé, 2000). During this period, life-history research gained prominence. These studies illuminated the complexity of disappearing or transforming professions, and emphasized the interplay between sociopolitical contexts, individual and collective practices, and identity formation in times of transition (Dubar, 2010; Muller Mirza & Alber, 2019).

Other research, inspired by Bruner and Vygotsky, examined how narration could serve as a developmental tool in professional support and training (Alheit et al.,^1995^). Storytelling was seen not only as a means of recording information but as a pathway to *agency*. These biographical approaches drew on two key intellectual traditions: first, the legacy of popular education, particularly the work of Paulo Freire; and second, the interpretive, interactionist, and ethnographic methodologies of the Chicago School of Sociology.

Freire’s contributions to adult learning research have been especially influential. In the 1940 s, tasked with increasing literacy among millions of rural Brazilians, Freire developed a pedagogy grounded in listening to learners’ lived experiences rather than delivering pre-designed curricula. Learners were not seen as passive recipients of knowledge, but as bearers of situated expertise. Knowledge was thus co-constructed through dialogue and rooted in the daily realities and struggles of learners. For Freire, literacy was not merely a technical skill, but a tool for transforming one’s understanding of experience and for uncovering mechanisms of social and symbolic domination. Though rooted in education, Freire’s ideas have important implications for developmental psychology, particularly in foregrounding the asymmetries and power relations inherent in learning relationships across the lifespan. His work highlights two central principles:

- Adult learning cannot be reduced to the acquisition of decontextualized content; it has developmental potential for both individuals and communities.
- Everyday experience constitutes a legitimate site of knowledge production, even in the absence of formal recognition or qualification.

The second major influence is the work of early sociologists from the Chicago School (W.I. Thomas, F. Znaniecki, H. Blumer, G.H. Mead, and later E. Goffman and H. Becker). In 1940 s, Chicago was a city marked by immigration, urban poverty, racism, and violence. These scholars developed innovative methodologies that gave voice to the lived realities of marginalized groups. They rejected the notion of fixed or objective social facts, emphasizing instead the situated and interactional nature of social life. Their work has deeply influenced psychology and education research, particularly concerning learning processes. Three key contributions can be highlighted from this body of work for our reflection: (1) Processes of personal and community transformation must be studied in their temporal trajectories and social contexts; (2) Researchers must take seriously the actor’s perspective and the meanings they attribute to their experiences. The object of learning is not accessible apart from how individuals express and interpret it; (3) Methodologically, this requires attention to everyday experiences and the use of “life documents” (e.g., letters, autobiographies, interviews) as valid sources for studying psychological and social processes from the actor’s point of view (Thomas & Znaniecki, 1918).

These insights have gone beyond sociology and expanded the territory of psychology and education, and reframed how adult learning is understood. Learning is no longer confined to formal schooling, and the boundary between scientific and practical knowledge is no longer essentialized (Muller Mirza & Perret-Clermont, 2016).

Experience itself becomes formative and transformative, particularly in contexts marked by uncertainty and change (Bruner, 1990; Dewey, 1934/1980; Muller Mirza & Tartas, 2023).

Several key insights emerge from this body of work:

- The epistemic value of *experience*: Experience is not merely raw data for reflection - it is a legitimate source of knowledge. Meaning-making in uncertain situations can lead to transformation under certain conditions.
- The role of *temporality*: These approaches highlight long-term trajectories, allowing researchers to trace how learning unfolds across different life stages and social contexts.
- *Narration* as a developmental tool: Life stories allow individuals to impose order, coherence, and meaning on their experiences. Narration is not only descriptive but performative, contributing to self-construction and subjectivity (Bruner, 2002).
- The identification of *learning experiences* as drivers of transformation: Life stories often reveal critical moments linked to transitions, ruptures, or intense social encounters that catalyze new understandings or ways of being.

Taken together, these biographical and narrative approaches offer rich theoretical and methodological resources for studying adult learning within a sociocultural framework in psychology of development. They foreground the dynamic interplay between personal history, social context, and meaning-making, highlighting how learning is embedded in people’s lives. They invite developmental psychologists to expand their scope beyond formal education and childhood, to include the diverse, situated, and dialogical ways in which adults continue to learn and grow throughout the lifespan (Tartas et al., 2023; Zittoun et al., 2013).

Nevertheless, certain limitations must be noted. By focusing on the singularity of biographical trajectories, some studies risk overlooking the broader historical and sociological processes that give meaning to these lives (Alhadeff-Jones, 2021). Moreover, the contributions of developmental psychology are often implicit. As a result, some studies document the complexity of life courses without fully exploring the psychosocial mechanisms that underlie transformation.

## A Sociocultural and Dialogical Approach to the Relationship Between Crisis and Learning Processes

The works of Lev Vygotsky (1934), Herbert Mead (1967), and their followers offer key contributions to the study of learning and its role in development across the life course, even though these authors rarely focused explicitly on adult learning. Their theories provide tools to transcend the binary between conceptions of individuals as either entirely shaped by external realities or entirely autonomous agents acting upon the world (Grossen, 2010; Valsiner, 2007; Zittoun, 2022). From a sociocultural perspective, thought is not confined to the mind or the brain but is situated and distributed across people, objects, and sociocultural contexts (Hutchins & Klausen, 1996). Humans are seen as creators and users of cultural tools within already-structured environments (Vygotsky, 1930/1985). Language plays a central role, not only as a tool for communication, but as a system of signs, a medium through which learning and psychological development occur (Vygotsky, 1934). Meaning-making processes form the foundation of thought. Humans are fundamentally meaning-makers, and personal meaning or sense is itself the product of social interaction. From this perspective, it is not isolated behaviors or internal cognitive structures that are the object of psychological study, but rather the processes of meaning construction and the transactions that shape them (Bruner, 1990).

Within this framework, the concept of *crisis* - broadly understood as a rupture - appears in contemporary research as a pivotal phenomenon. Crises are often interpreted by individuals as disruptions in their environments and can open pathways for transition, reflection, and development (Zittoun, et al. this issue).

### The Study of Social Interactions for Understanding Learning Processes

Zooming in on learning processes more specifically, it is worth recalling that rupture and contradiction were central concepts for Vygotsky in his effort to develop a “paedological” theory of development. These ideas are fundamental to his dialectical conception of thought, which posits an ongoing tension between inner and outer worlds. According to Vygotsky, it is through confrontation with the cultural environment that new psychological functions emerge: “from a real conflict between organism and environment and from active adaptation to the environment” (Vygotsky, 1931/1974, p. 191). In this perspective, school becomes essential as a space that constitutes a rupture with everyday life, allowing students to elaborate scientific concepts.

By “environment,” Vygotsky refers to the historical and cultural context, where culture is the product of social life and humans’ activities (Schneuwly, 1994). Human action is always mediated by tools that are socially developed. Transformation occurs through the use of these tools. The shift from external to internal – what Vygotsky called internalization – happens through the mediation of sign systems, especially language:

“words initially exist objectively for others, and only afterwards begin to exist for the child himself” (Vygotsky, 1931/1974, p. 201).

Building on this legacy, a significant body of research has examined the role of language in learning interactions, especially in classrooms (Grossen & Muller Mirza, 2019). Studies have shown that dialogical practices – such as exploratory talk (Mercer & Littleton, 2007) and argumentation (Muller Mirza & Perret-Clermont, 2009) – often involve contradictions and ruptures in the flow of thought and play an important role in knowledge construction. These “conflictual” interactions, however, are not in themselves learning factors. Rather, what matters are the interactional dynamics, the construction of intersubjectivity (Grossen, 2010; Grossen & Muller Mirza, 2020), and what we term *dialogical tensions*.

### Dialogues and Dialogical Tensions

The concept of dialogical tension, drawing on the work of Bakhtin/Voloshinov (1986) and contemporary scholars, has primarily been applied to studies of educational interactions (Grossen & Muller Mirza, 2019) and professional contexts such as therapeutic interviews (Grossen & Salazar Orvig, 2011). Grossen and Salazar Orvig, for example, demonstrated that the voices evoked by interlocutors – past, present, imagined – serve as discursive resources.

In certain contexts, such as intercultural education lessons that involve emotionally charged personal experiences, these voices from other times and places may complicate learning, particularly when learners struggle to reframe lived experience into new conceptual categories (Grossen & Muller Mirza, 2020; Muller Mirza, 2023; Pramling & Säljö, 2014; Zittoun & Grossen, 2013).

We believe this notion is particularly useful for analyzing lifelong learning and crisis, especially when grounded in dialogical theories of the self (Linell, 2009; Marková, 2016). Such frameworks allow us to study the meaning (which integrates cognitive and emotional processes) that individuals attribute to their experiences, the contradictions or ruptures they identify, and the ways in which they semiotically elaborate these tensions in their relational, social, and cultural contexts (Valsiner, 2005).

By starting from the individual’s orientation toward the other – the fundamental interdependence between self and other, and thus the intersubjective nature of the psyche – this approach prevents us from treating crises as purely external events that cause internal reactions. Instead, it brings into focus the social dimensions of subjectivation – the processes through which persons come to define themselves as unique. As Grossen writes:

“Dialogism leads us to move beyond the immediacy of a given situation by considering both the temporal dimension and the fact that every individual is engaged in multiple spheres of experience”, and that “through imagination, individuals may revisit the past and project themselves into the future” (Grossen, 2021, p. 33).

Articulating dialogical approaches to the study of human thought thus challenge solipsistic, linear models of cognition. They conceptualize thinking as a process of meaning-making that is fundamentally dialogical, where the other is not external but constitutive (Marková, 2006, 2016). The psyche is understood as a tension-filled dialogue between *I* and *you*, here and elsewhere, past, present, and the imagined future (Muller Mirza & dos Santos Mamed, 2021). Here, dialogue refers not only to face-to-face interaction, but more broadly to the idea that.

“any discourse (even a dialogue with oneself) echoes the voices of discourses that were held elsewhere at other times and in other situations” (Grossen & Salazar Orvig, 2011, p. 494).

Dialogue is no longer merely a tool for constructing knowledge; rather, the self and reality are constituted in and through dialogue. Verbal interaction is thus seen as a layered network of present and absent dialogues.

Research in this field has operationalized different dimensions of dialogicality. In the dialogical analysis of biographical narratives, Grossen (2015) identifies three distinct types of dialogue:

- Dialogue at a distance: language is crossed by the voices and discourses of absent others;
- Dialogue across situations: references are made to other times, places, or contexts (cf. Linell’s concept of “intersituational” dialogue);
- Autodialogism: in autobiographical or diary writing, the narrator is both storyteller and recipient, addressing others and herself. This concept refers to the presence of multiple voices within a single person’s discourse, which may generate more or less pronounced dialogical tensions.

This typology provides a useful analytical framework for the methodological reflection that follows.

### Synthesis and Directions for Analyzing Learning Across a Life Trajectory

The conceptual framework outlined above leads to several theoretical and methodological implications for studying learning over the life course. It highlights the dynamic, processual, and dialogical dimensions of adult learning, as well as the critical role of narration – both for the *writer*, who uses the narrative to document lived experiences, salient moments, and everyday routines, and to reflect on personal development and learning, and for the *researcher*, who gains access to a rich dialogical material over the time.

More specifically, this framework supports the hypothesis that autobiographical material, especially written forms such as diaries, must be approached with attention to the *semiotic* dimension (i.e., how experiences are put into words) and to two key analytical aspects: the multiplicity of *voices* engaged in the narrative, and the *tensions* or contradictions that may emerge between these voices, situated across different times and relational contexts.

These considerations led us to work with a narrative-based corpus in order to examine the relationships between crisis and learning across the life span.

## Analyzing a Life Narrative Through Dialogical Tensions

### The Specificity of the Data: A Personal Diary

The data consist of a personal diary authored by a woman, aged 50, whom we refer to here as *Applegirl*. She began keeping a diary at the age of 11, and we have obtained her consent to analyze the portion of her writing that was publicly shared on platforms such as Presebox between 2001 and 2021. This segment of her diary comprises approximately 730,000 words, the equivalent of around 3,000 pages. Writing plays a central role in her life. In one entry, she states:*“Writing has kept me sane”* (13 November 2020).

Writing a diary involves putting one’s lived experience into words – semiotizing it, in Valsiner’s terms (2005; 2007) – and doing so offers not only a means of constructing and reconstructing memory, but also of pausing the flow of life in order to reflect. The diary is not the experience itself but a written text through which the narrator puts her experience into words and which shapes the frame of her experience (Grossen, 2015).

A diary is rarely strictly private. Even when written primarily for oneself, as Lejeune (2003) reminds us, diarists may end up publishing their texts or secretly hope to do so. In the case examined here, Applegirl’s diary occupies a hybrid position: it is written mainly for herself, yet it is also made public through online platforms that allow other users to read, comment on, and engage in dialogue with the author.

This constitutes what Grossen (2015) has described as a “personal-public diary.” Her writing often addresses a potential audience and explicitly responds to the voices she evokes, family members, colleagues, but also institutions, authorities, media, and broader social discourses. In this way, others are both the *source* and the *horizon* of her narrative.

Her diary makes visible both *external* dialogues (with absent or imagined interlocutors) and *internal* dialogues (with herself, in the form of questions, doubts, and imagined counter-voices). These data are thus particularly valuable for three reasons:

- They reflect *experiences* that are meaningful to the narrator – not as raw events, but as interpreted and emotionally charged;
- They span a *long temporal range*, making it possible to trace both personal transformations and changes in the broader environment;
- They reveal *movements of thought and dialogical tensions* that point toward processes of meaning-making and potential development.

### Method of Analysis

The analysis followed a multi-step methodology (inspired by Zittoun & Gillespie, 2012 and Zittoun, et al., 2024):

1. Initial screening of the diary was conducted with the support of Python-based text analysis tools, using a predefined list of keywords related to learning situations or transitions (e.g., “learning,” but also broader terms reflecting change, reflection, or questioning). This made it possible to identify:
  Learning domains (themes or areas of experience that involve the acquisition or transformation of knowledge);
  Episodes or “scenes” in which the narrator explores her experience reflexively.
2. Based on this screening, a visual mapping of the data was created to identify key moments. The function of “intensity” (e.g., frequency of learning-related terms, presence of emotional or evaluative language) provided by Python proved helpful in selecting significant posts that met our analytical criteria.
3. Once the most relevant segments were identified, a qualitative, fine-grained analysis was conducted to identify *dialogical tensions*, that is, moments when different voices, perspectives, or value systems come into contact or conflict, producing potential conditions for reflection and transformation.

Our research questions are the following:

- Which domains and learning experiences appear in Applegirl’s narrative, particularly between 2001 and 2021?
- How do dialogical tensions appear in her writing, and what roles do they play – as catalysts or obstacles – in processes of learning and development?
- To what extent do these learning experiences serve as opportunities for psychological development, understood as the reconfiguration of experience? Under which conditions does this occur?

## Findings – Learning Domains, Dialogical Tensions, and the Conditions for Thought

### Learning Domains in Applegirl’s Life Trajectory (2001–2021)

Applegirl is a woman in her fifties who has been married for over thirty years. Her two children, a daughter and a son, have left home, and she now resides in a town near the Great Lakes in the northern United States. Coming from a modest background, and being the only one among her siblings to attend university, Applegirl pursued a degree in literature. Her professional and personal trajectory reflects a rich and multifaceted life course: she began working in a restaurant, eventually became a manager after receiving on-the-job training; she later dedicated herself to homeschooling her children; she engaged in missionary work with a non- governmental organization in several countries in the Global South; and eventually secured a teaching position in her hometown, where she still works today. During the COVID-19 pandemic - a period during which many people’s professional activities were abruptly suspended - she chose instead to deepen her professional engagement.

In this brief, factual summary, centered on situations where learning, teaching, and training were prominent, Applegirl’s life story illustrates a contemporary trajectory of a woman shaped by transitions, discontinuities, reskilling periods, paid and unpaid labour, and the construction and mobilization of competencies across seemingly disparate domains. This narrative invites questions about the role of family dynamics, especially significant in her life as a woman, in shaping decisions to enter, leave, or change professional pathways, all within a broader sociohistorical context. However, such a summary inevitably omits the texture of lived experience: the doubts and hesitations she may have faced before accepting a managerial role that distanced her from her children; the motivations, both explicit and implicit, behind her decision to homeschool; the moments of joy described as “life experiences,” during travel, teaching, and community involvement; the illnesses and family conflicts; the episodes of sadness that may be read as depressive symptoms; and the symbolic resources and mediating figures that shaped her subjectivity.

Interestingly, Applegirl’s learning experiences appear to be closely linked to life transitions: becoming a mother, changing careers, navigating family conflict, and undergoing bodily transformations. These experiences contribute to a gradual *redefinition of her worldview*, from one oriented toward the past, marked by guilt and sadness linked to a difficult childhood, toward a position more grounded in the present, more resilient, and focused on self-care, care for others, and engagement with her immediate environment. Yet, her diary also contains sustained reflections and doubt, which remain a constant feature of her narrative voice.

Several cross-cutting topics appear throughout her learning trajectory and emerge as particularly salient at certain life moments, functioning as *sites of dialogical tension*: conflicts with family members (particularly with her father, with whom she cuts ties in 2014), financial hardship within her marriage, and various physical and psychological health issues. What seems to provide continuity across her trajectory are the resources she draws upon, which support both her learning and her resilience. These include:

- *Creativity* in the face of adversity: for example, developing a family garden modeled on the book “Animal, Vegetable, Miracle: A Year of Food Life” (Kingsolver, 2007) to reduce costs and generate income; designing an emergency fund after the 2008 financial crisis to cope with household debt; and inventing educational games and pedagogical activities while homeschooling her children.
- *Curiosity* about the world: expressed through hosting international students, traveling abroad, and participating in NGO work in the Global South.
- *Faith*: which evolves over time, from a deep religious commitment within her church community to a more critical, personal spirituality that eventually distances itself from religious dogma.

Despite occasional tensions in her marriage, her husband consistently appears in the diary as a figure of tenderness, support, and shared decision-making. Her children represent, throughout her life, a central anchor of identity. Around 2019, coinciding with her children leaving home and a difficult period marked by unresolved health and financial issues, she begins therapy. Writing in her journal becomes, definitively, a lifeline: a tool for survival and psychological continuity.

### Dialogical Tensions in the Narrative

In this section, we focus the analysis more precisely in order to test the analytical tools of dialogical inquiry, examining a series of posts that focus on specific activities. The challenge of this analysis was to delimit a segment of the narrative that would allow us to fully develop our dialogical approach. In a broader analysis, we decided to follow the theme of *work*, both paid and unpaid, across the entire diary to better understand how tensions, transitions, and opportunities for learning emerge and evolve in everyday life.

The *learning* described in these posts, whether through formal training or lived experience, proves substantial. Across her diary, Applegirl repeatedly reflects on her professional decisions, expressing dilemmas, doubts, frustrations, and emotional struggles linked to work choices and transitions. These moments provide fertile ground for identifying different types of dialogical tension (with self, others, or broader normative contexts), as well as their potential impact on learning and development.

For this article, we focus on four posts written between 2002 and 2006, a critical period in Applegirl’s life during which she actively questions her professional future, makes a commitment to further engage in her chosen career, and then ultimately decides to leave it.

Two to three years after completing her university degree, Applegirl is hired by a restaurant chain in 2004. Over time, she develops a strong sense of belonging to the organization, to the extent that her chosen pseudonym on public platforms aligns with the company’s name. In 2005, she decides to enroll in a training program for managers. She views herself – and is seen by others – as highly competent. Nonetheless, she perceives the training as necessary to complete her professional knowledge. After completing the program, she is promoted to a managerial position. Yet only a few months later, she chooses to leave her job to devote herself to her children’s education.

These posts offer rich material for analyzing three levels of dialogical interaction:

1. Dialogue with the *norms*, values, or broader sociocultural contexts, including social discourses about motherhood, work, and success;
2. Dialogue with significant *others*, such as family members, colleagues, or supervisors;
3. Dialogue with *oneself*, often expressed through questions, self-doubt, and internal debate.

These levels of dialogue do not function in isolation; rather, they interact and sometimes conflict, creating tensions that may catalyze transformations in identity, perspective, and meaning. From a dialogical perspective, these tensions in discourse are intimately linked to tensions in thought, and it is precisely through this entanglement that learning and development become possible.

Excerpt 1 – June 27, 2002

Another thing I’ve been thinking about is what I want to do with my life. I want to get a degree, any degree. SO, since I am closest to my English degree, I will pursue that. But what next? Substitute teach the rest of my life? Or go back to school? And if I go back to school I want to check into getting a degree in physical therapy or nutrition or something. I think it would be rewarding, it would be good for me to keep on track and I am very interested. I just wonder how much schooling it involves… I will have to get more info.

This entry, written at a time when Applegirl is married and has recently given birth to her second child, yet has not completed her studies, is marked by the repetitive use of “I” and a series of exploratory utterances that reflect an inner dialogue. The writer engages in self-questioning (*“what I want to do with my life”*, *“But what next?“*), suggests tentative answers (*“I want to get a degree”*), examines options (*“Or go back to school?“*), and formulates hypotheses (*“I think it would be rewarding”*) as well as future intentions (*“I will have to get more info”*).

This inner dialogue is also shaped by implicit references to broader social discourses. The entry reveals the influence of cultural norms that associate higher education with personal success and economic mobility (*“get a degree*,* any degree”*). Although not directly invoked, one can infer the presence of significant others – perhaps educators, family members, or peers – whose values and expectations subtly inform Applegirl’s thought process. These implicit voices form part of the dialogical context in which her reflections take place.

At the heart of this excerpt lies a dialogical tension between personal motivation (*“I am very interested”*) and pressures to conform to societal or familial expectations, such as financial security or efficient career planning (*“any degree*,*” “keep on track”*). These tensions make her decision-making process uncertain and fragmented. What is especially notable here is how thought itself takes a dialogical form: language becomes the medium through which alternatives are weighed, doubts expressed, and possibilities tested.

Excerpt 2 – January 23, 2004

Still Alive, Frozen, But Alive. Work is going weird. I would say that I am one of the most responsible and reliable people there. Besides my regular duties, I do the To Go and Host scheduling, I am a trainer and the lead trainer at that, I am a key and they just ordered me an assistant manager name tag. I don’t feel nearly trained enough to do the job of a key, let alone an assistant manager. I mean, I can pretty much use the computer, cut the floor and deal with guests, but I know NOTHING about the workings of the back of the house, nothing about food prep or cooking, and almost nothing about managerial paperwork, etc. […] I wish I had more training so that I could learn more about what I am supposed to be doing when I manage/key. I guess right now my two advantages are that I am smarter than this other girl (not bragging, just stating facts) and that I have a more normal life (she is a little screwed up personally living in hotels with her kids, cheating on her long-time boyfriend, etc… weird) and the other advantage I have is that Walter’s boss REALLY likes me… and I’ve worked for Applebees longer than her. I guess she really really wants this managing position, and she says that it would be like a real career move for her. I feel like it would be nice to have a steady salary and benefits, but I don’t think this would be my end-all-perfect-ideal career, do you know what I am saying? At the same time, i think I would do a great job, and I am definitely a leader.

Two years later, Applegirl reflects on her evolving role at work. While fewer interrogative forms are used compared to the earlier entry, the narrative still exhibits a dialogical structure. Expressions of uncertainty (*“I guess*,*” “I don’t feel*,*” “I don’t think”*) are counterbalanced by affirmations of competence (*“I am smarter*,*” “I am definitely a leader”*). What emerges is a dynamic interplay between a present self, who questions and evaluates, and a future or potential self, the “manager-in-becoming.”

This inner dialogue is framed by explicit references to others: coworkers, supervisors, and particularly a rival colleague. Social comparisons and perceived judgments from managerial figures (*“Walter’s boss REALLY likes me”*) serve to reinforce her self-positioning. These external voices are not merely background; they actively participate in shaping Applegirl’s sense of professional legitimacy.

The dialogical tension here lies between a perceived lack of technical competence (due to insufficient training) and a growing sense of leadership and experience. Another layer of tension is found in the contrast between Applegirl’s personal preferences (*“I don’t think this would be my end-all-perfect-ideal career”*) and the practical appeal of stability (*“steady salary and benefits”*) – a concern likely rooted in familial responsibilities. This tension between institutional discourses of professional success and subjective self-construction reflects how identity development is negotiated dialogically across competing values and voices.

Excerpt 3 – February 10, 2006

Bundled up to go sledding… Conclusion. I have officially come to the conclusion that my children are infinitely more important than my career or a job promotion.

I will be speaking with my GM about keeping my 4-day weeks and not being promoted into the KM or GM position until my kiddos are both in to school full time.

I really want to spend that year with Jonah when Mattie is in school full time next year. He never had time alone with me and it will be great for him. I can always work on me eventually.

In this entry, written two years after the previous one, Applegirl adopts a more assertive tone. The title - *Conclusion* - and the declarative structure suggest that an inner negotiation has. reached closure. The dialogical dynamic is subtler but still present, operating in the background as a completed reflection. She appears to be reaffirming a decision made earlier, possibly as a form of self-justification.

This moment marks a shift: from career achievement, previously portrayed as desirable if not ideal, to parental commitment as the primary axis of identity. Applegirl’s re-evaluation of her professional aspirations (e.g., declining promotion opportunities) represents a significant transformation, not necessarily as a renunciation but rather as a reconfiguration of priorities. The implicit contrast with her earlier ambitions in health-related fields (*“I think it would be rewarding”*) suggests an ongoing dialogue with past versions of herself.

The most salient dialogical tension here is between professional ideals and parental ideals, which are no longer in competition but are now subordinated to one another. The voices of her children, though not speaking directly, occupy an emotionally charged position in the narrative (*“infinitely more important*,*” “I really want to spend that year with Jonah”*) and guide the decision-making process. The presence of her partner is implied, and her decisions seem to reflect a family-centered frame of reference.

Excerpt 4 – April 14, 2006

School. Tim and I had an awesome talk today about the kids. I have kicked around the idea of home- schooling both Mattie and Jonah since I was pregnant with Mattie. Tonite, he was much more supportive and enthusiastic than ever…I think because the reality of sending our kids to school is hitting him with Mattie going to kindergarten round up… I am very willing to do this, I just need some enthusiasm and support from him.

I think we are going to take the time to really research it, talk with some other families. and do it if it fits our needs.

Two months later, Applegirl documents a key conversation with her husband regarding the possibility of homeschooling their children. This entry shifts from an individual “I” to a collective “we,” marking the emergence of a dialogue within the family sphere as central to her meaning-making. She explores her own position (*“I am very willing*,*” “I just need”*) while also hypothesizing about her partner’s thoughts and motivations (*“I think because the reality is* hitting him”).

This interplay of voices – her own, her husband’s, and those of unnamed other families they plan to consult – creates a multi-layered dialogical space. The act of deliberating whether homeschooling would “fit our needs” signals an ongoing process of collaborative sense- making.

The tension here arises between personal intention, the need for relational support, and implicit societal norms about formal education. Applegirl is not only negotiating with her partner but also with broader cultural expectations regarding schooling and the mother’s role within it. The decision to homeschool is presented as both a potential rupture with dominant norms and a continuation of deeply felt family values.

## Discussion: Dialogical Tensions and Learning Across Time

These four entries, written over four years, offer an illustration of dialogical tensions, especially in contexts involving learning, career, and parenting. The diary functions as a “dialogical space” (Grossen, 2015) where social norms, significant others, and internal voices interact, sometimes in harmony, often in tension. Importantly, these tensions are not indicative of dissonance or paralysis but rather constitute the very mechanism through which identity and meaning evolve.

Analysing such longitudinal data reveals the temporal layering of thought and self-positioning. Early aspirations are revisited, transformed, or displaced by later commitments. Crises – whether implicit or explicit, social or personal – serve as catalysts for reconfiguration of the self rather than mere disruption.

Such an analysis invites us to consider diaries not simply as reflective tools, but as sites of semiotic mediation and dialogical tensions, where individuals navigate and actively construct their place within overlapping spheres of experience, personal, relational, and societal. It also deepens our understanding of how learning is embedded in key moments of personal trajectories, revealing that it cannot be reduced to the acquisition of formal knowledge.

## Conclusion

This paper has proposed an integrated theoretical and methodological framework to investigate the relationship between learning and crisis over the life course, grounded at the intersection of biographical and narrative approaches, and sociocultural and dialogical perspectives. By bridging these traditions, we aimed to capture the complexity and multidimensionality of learning as it unfolds in lived experience, particularly in moments of rupture, reorientation, or tension.

Theoretically, this work contributes to a growing body of research that seeks to move beyond static, linear, or institutionally bound models of adult learning. It does so by positing learning as a situated, semiotically mediated, and dialogically structured process, in which crises act not merely as disruptions but as potential *catalysts* for new forms of meaning-making. Drawing from a dialogical epistemology, we argued that thought and development emerge through the interplay of multiple voices, personal, relational, and societal, within and across time.

Our results demonstrate that learning is not confined to formal educational contexts, nor can it be understood in isolation from the broader cultural, relational, and material conditions in which it takes place. The transitions described in Applegirl narrative (her ambivalence toward career paths, her evolving parental commitments, her consideration of homeschooling) are all shaped by social norms (namely related to the place of the women and the men in the horizon of the contemporary life), relational dynamics, and historical moments. Yet they are also actively interpreted and reworked in a narrative process that mobilizes different forms of agency and imagination.

One of the central contributions of this study is to propose a nuanced understanding of crisis not as a purely subjective disruption, nor as a purely objective event, but as a privileged analytical entry point for exploring the dynamic relationship between inner life and outer realities. Crises – whether large-scale (economic collapses, pandemics) or biographical (transitions in work or family roles) – are phenomena that produce effects both on collectives and individuals. What our dialogical approach allows us to examine is how such events are made meaningful in and through narrative: how they are interpreted, negotiated, and integrated into evolving self-understandings.

Through the close analysis of a diary, we were able to trace how external constraints and social expectations were not simply endured or resisted but *refracted* through a dialogical process of sense-making, where the self is continuously redefined in relation to others, to social norms, and to past and future selves.

Methodologically, we have shown how autobiographical data, such as long-term diary writing, can serve as a valuable resource for accessing the “semiotised experience” of learning and transformation. Our analysis made visible the tensions between different voices and positions that coexist within personal narratives, revealing how learning often takes the form of negotiating meaning across multiple spheres of experiences, at the micro-, meso- and macro- levels.

More broadly, this work contributes to the field of human development by emphasizing the fundamentally dialogical nature of developmental processes. It affirms that subjectivity or self is always relational and situated; that learning occurs at the intersection of personal histories and collective contexts; and that development can be studied as a movement across tensions – between inner and outer worlds, between stability and change, between the familiar and the uncertain. It suggests that crises, far from being merely moments of rupture, may serve as critical zones of transformation, where individuals reconfigure their relationship to self, others, and the world.

While this study focused on a limited selection of narrative segments, it opens avenues for future analyses on longer and more diverse life trajectories. Analysing these moments through dialogical and narrative lens offers a powerful lever for understanding how people learn, adapt, feel joy or worry, and develop meaningfully throughout life.

## Data Availability

No datasets were generated or analysed during the current study.
